# Supplementary material for: Deep Transcriptome Analysis Reveals Reactive Oxygen Species (ROS) Network Evolution, Response to Abiotic Stress, and Regulation of Fiber Development in Cotton
Source: Int J Mol Sci. 2019 Apr 15;20(8):1863. doi: 10.3390/ijms20081863 (PMC6514600; doi:10.3390/ijms20081863)
Supplement: Supplementary file 1 [file ijms-20-01863-s001.zip › Supplementary files/Figure S1.pdf]

Supplementary Figure 3 GO enrichment analysis of M85 and CRI12 under alkaili-salt stress. the most enriched GO terms were indicated in A-L

Figure legends of DEGs enrichment analysis.

Figure	Species	Tissue	Time point post to treatment
A	M85	Leaf	3h
B	M85	Leaf	12h
C	M85	Leaf	48h
D	M85	Root	3h
E	M85	Root	12h
F	M85	Root	48h
G	CRI12	Leaf	3h
H	CRI12	Leaf	12h
I	CRI12	Leaf	48h
J	CRI12	Root	3h
K	CRI12	Root	12h
L	CRI12	Root	48h

A

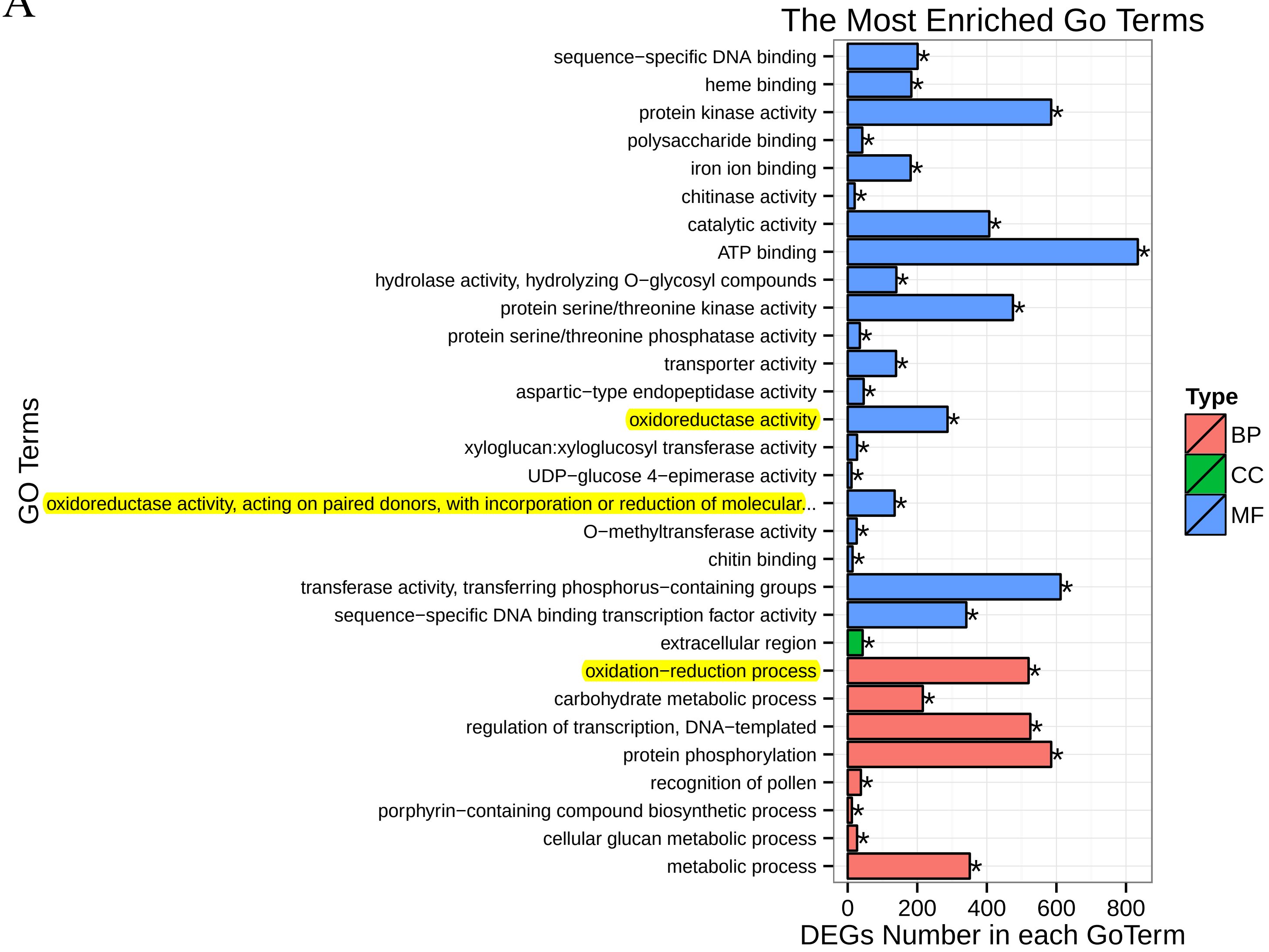

The Most Enriched Go Terms
GO Terms
sequence−specific DNA binding
heme binding
protein kinase activity
polysaccharide binding
iron ion binding
chitinase activity
catalytic activity
ATP binding
hydrolase activity, hydrolyzing O−glycosyl compounds
protein serine/threonine kinase activity
protein serine/threonine phosphatase activity
transporter activity
aspartic−type endopeptidase activity
oxidoreductase activity
xyloglucan:xyloglucosyl transferase activity
UDP−glucose 4−epimerase activity
oxidoreductase activity, acting on paired donors, with incorporation or reduction of molecular...
O−methyltransferase activity
chitin binding
transferase activity, transferring phosphorus−containing groups
sequence−specific DNA binding transcription factor activity
extracellular region
oxidation−reduction process
carbohydrate metabolic process
regulation of transcription, DNA−templated
protein phosphorylation
recognition of pollen
porphyrin−containing compound biosynthetic process
cellular glucan metabolic process
metabolic process
0
200
400
600
800
DEGs Number in each GoTerm
Type
BP
CC
MF

B

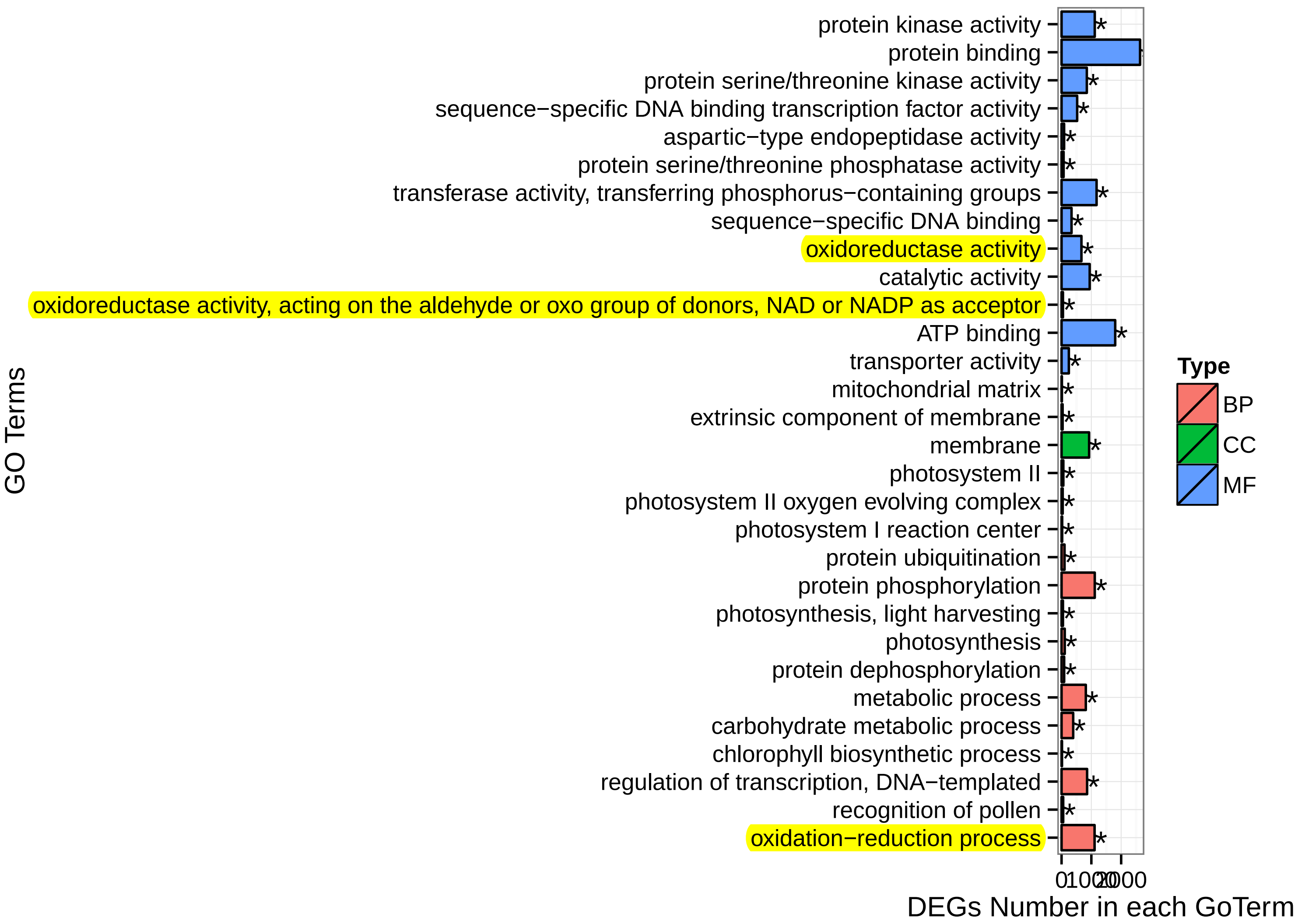
protein kinase activity
protein binding
protein serine/threonine kinase activity
sequence−specific DNA binding transcription factor activity
aspartic−type endopeptidase activity
protein serine/threonine phosphatase activity
transferase activity, transferring phosphorus−containing groups
sequence−specific DNA binding
oxidoreductase activity
catalytic activity
oxidoreductase activity, acting on the aldehyde or oxo group of donors, NAD or NADP as acceptor
ATP binding
transporter activity
mitochondrial matrix
extrinsic component of membrane
membrane
photosystem II
photosystem II oxygen evolving complex
photosystem I reaction center
protein ubiquitination
protein phosphorylation
photosynthesis, light harvesting
photosynthesis
protein dephosphorylation
metabolic process
carbohydrate metabolic process
chlorophyll biosynthetic process
regulation of transcription, DNA−templated
recognition of pollen
oxidation−reduction process
GO Terms
0
1000
2000
DEGs Number in each GoTerm
Type
BP
CC
MF

C

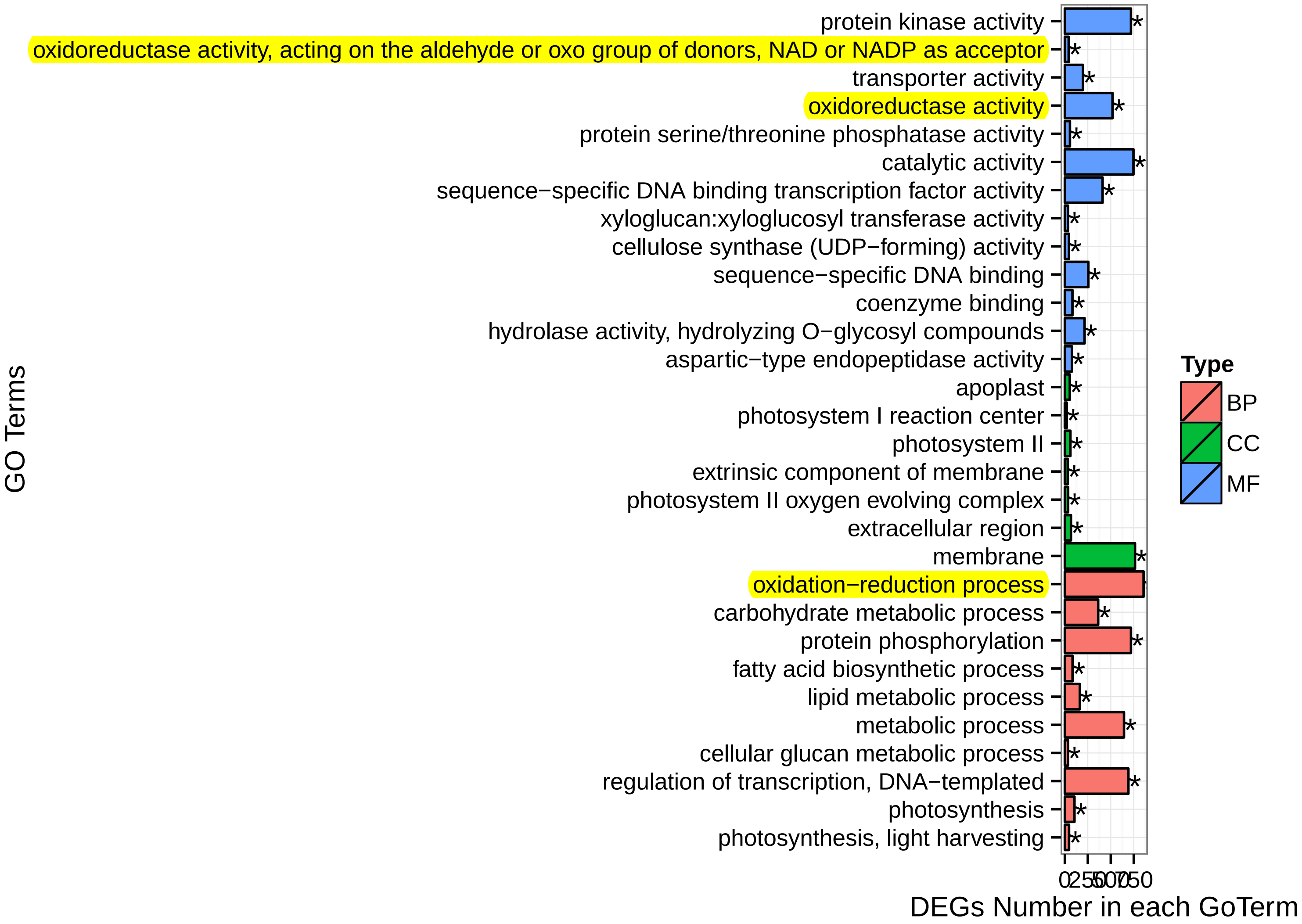
protein kinase activity
oxidoreductase activity, acting on the aldehyde or oxo group of donors, NAD or NADP as acceptor
transporter activity
oxidoreductase activity
protein serine/threonine phosphatase activity
catalytic activity
sequence−specific DNA binding transcription factor activity
xyloglucan:xyloglucosyl transferase activity
cellulose synthase (UDP−forming) activity
sequence−specific DNA binding
coenzyme binding
hydrolase activity, hydrolyzing O−glycosyl compounds
aspartic−type endopeptidase activity
apoplast
photosystem I reaction center
photosystem II
extrinsic component of membrane
photosystem II oxygen evolving complex
extracellular region
membrane
oxidation−reduction process
carbohydrate metabolic process
protein phosphorylation
fatty acid biosynthetic process
lipid metabolic process
metabolic process
cellular glucan metabolic process
regulation of transcription, DNA−templated
photosynthesis
photosynthesis, light harvesting
GO Terms
0
250
500
750
DEGs Number in each GoTerm
Type
BP
CC
MF

D

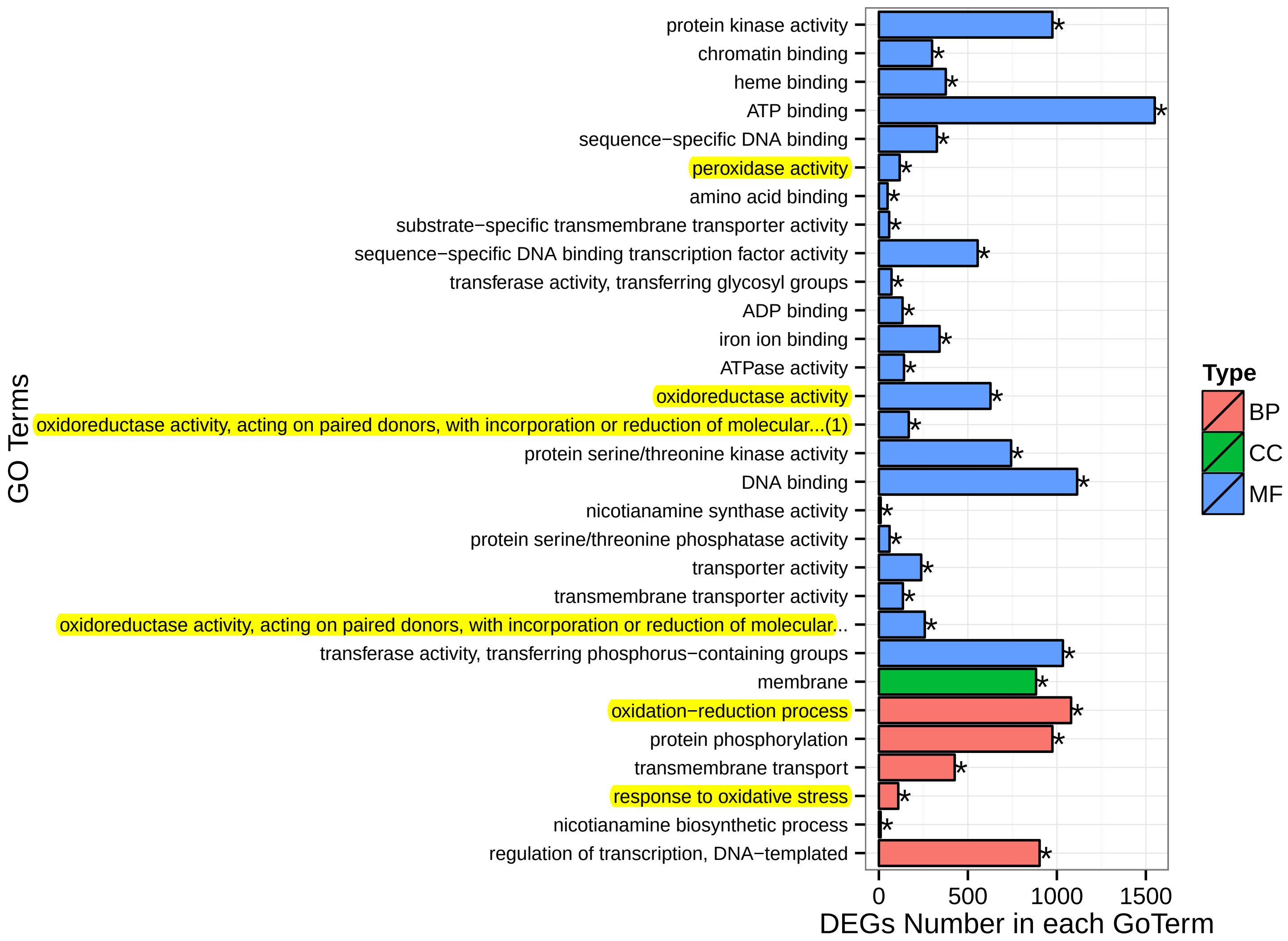
GO Terms
protein kinase activity
chromatin binding
heme binding
ATP binding
sequence−specific DNA binding
peroxidase activity
amino acid binding
substrate−specific transmembrane transporter activity
sequence−specific DNA binding transcription factor activity
transferase activity, transferring glycosyl groups
ADP binding
iron ion binding
ATPase activity
oxidoreductase activity
oxidoreductase activity, acting on paired donors, with incorporation or reduction of molecular...(1)
protein serine/threonine kinase activity
DNA binding
nicotianamine synthase activity
protein serine/threonine phosphatase activity
transporter activity
transmembrane transporter activity
oxidoreductase activity, acting on paired donors, with incorporation or reduction of molecular...
transferase activity, transferring phosphorus−containing groups
membrane
oxidation−reduction process
protein phosphorylation
transmembrane transport
response to oxidative stress
nicotianamine biosynthetic process
regulation of transcription, DNA−templated
0
500
1000
1500
DEGs Number in each GoTerm
Type
BP
CC
MF

E

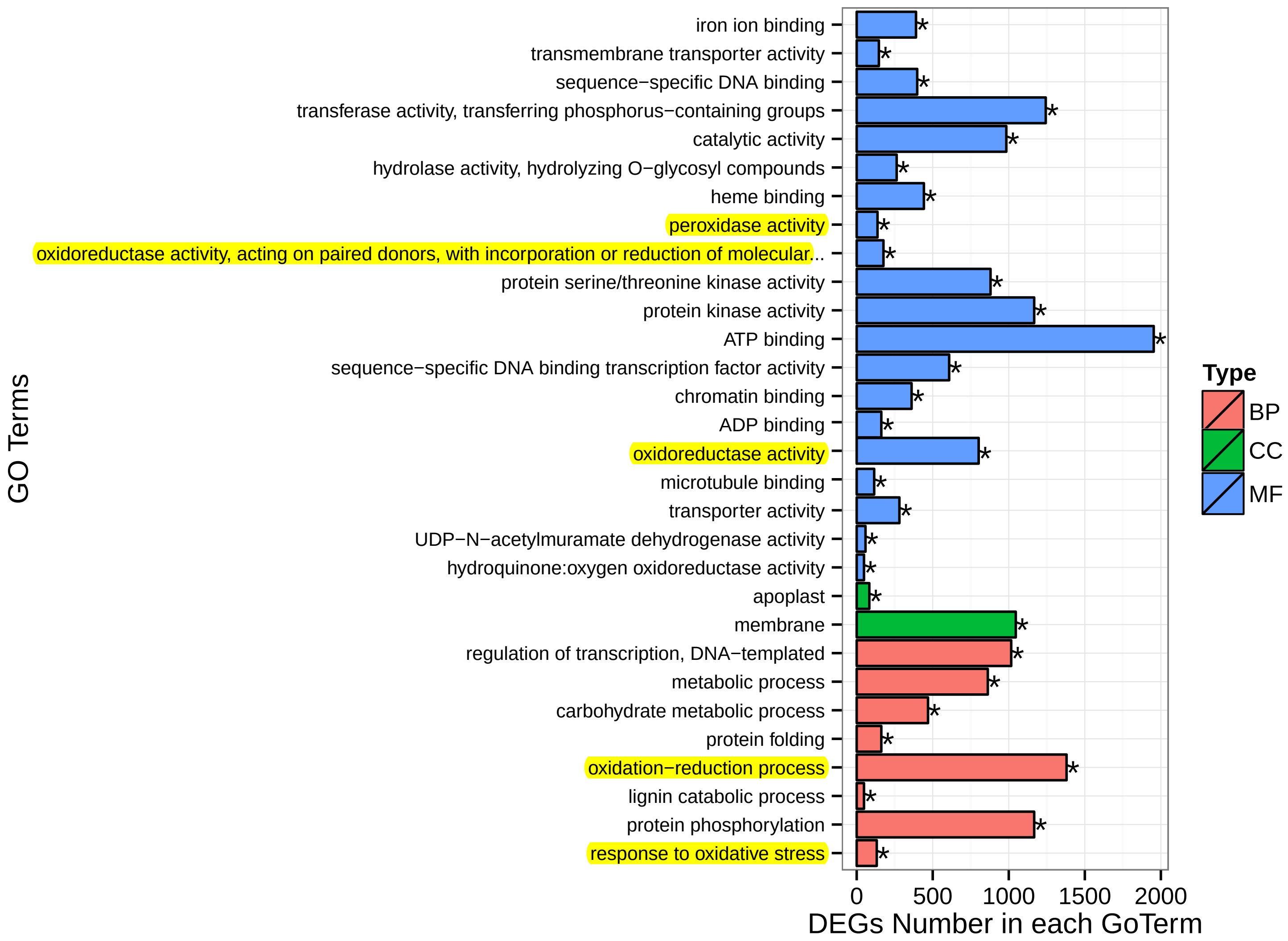
iron ion binding
transmembrane transporter activity
sequence−specific DNA binding
transferase activity, transferring phosphorus−containing groups
catalytic activity
hydrolase activity, hydrolyzing O−glycosyl compounds
heme binding
peroxidase activity
oxidoreductase activity, acting on paired donors, with incorporation or reduction of molecular...
protein serine/threonine kinase activity
protein kinase activity
ATP binding
sequence−specific DNA binding transcription factor activity
chromatin binding
ADP binding
oxidoreductase activity
microtubule binding
transporter activity
UDP−N−acetylmuramate dehydrogenase activity
hydroquinone:oxygen oxidoreductase activity
apoplast
membrane
regulation of transcription, DNA−templated
metabolic process
carbohydrate metabolic process
protein folding
oxidation−reduction process
lignin catabolic process
protein phosphorylation
response to oxidative stress
GO Terms
0
500
1000
1500
2000
DEGs Number in each GoTerm
Type
BP
CC
MF

F

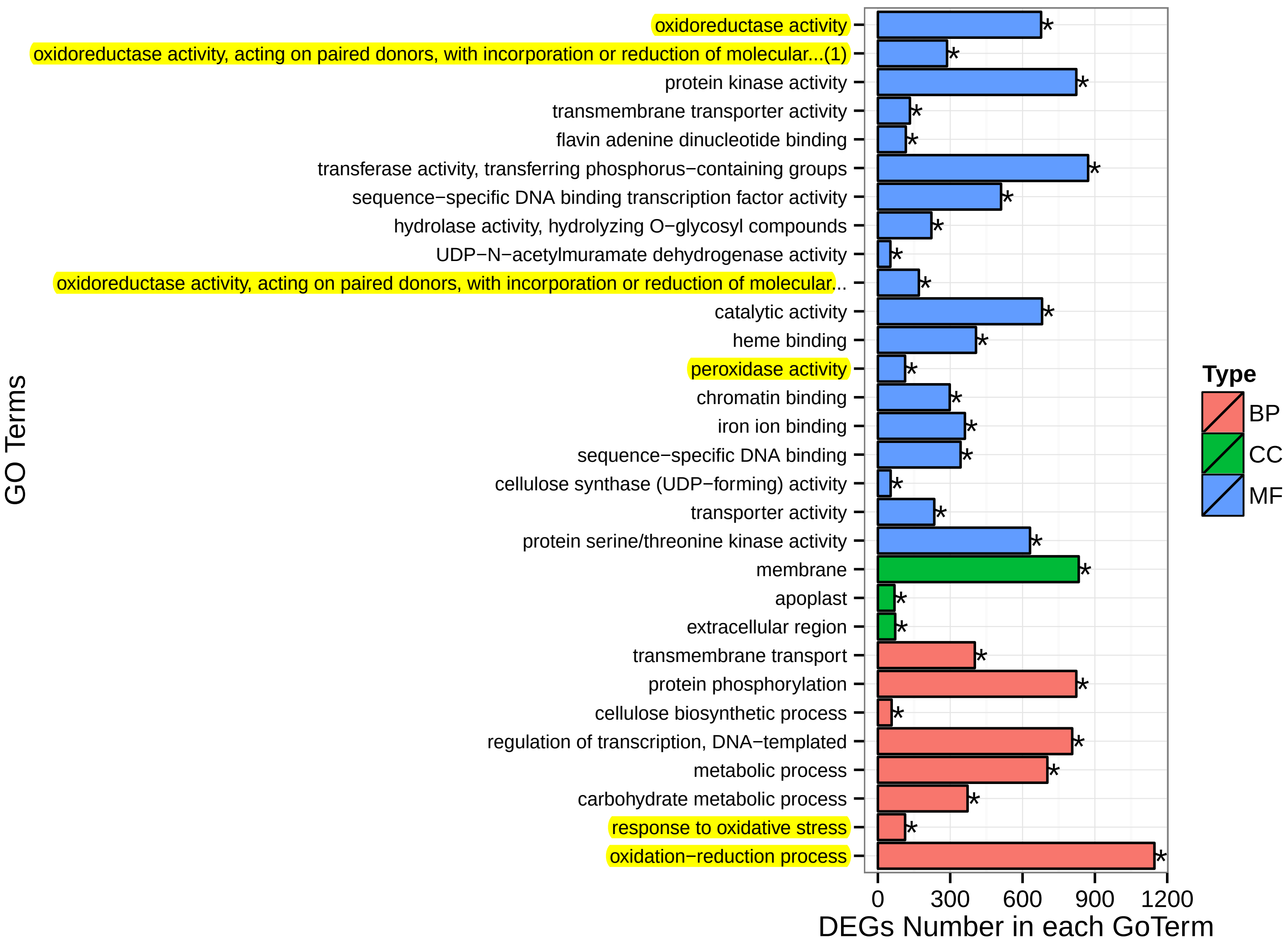

oxidoreductase activity
oxidoreductase activity, acting on paired donors, with incorporation or reduction of molecular...(1)
protein kinase activity
transmembrane transporter activity
flavin adenine dinucleotide binding
transferase activity, transferring phosphorus−containing groups
sequence−specific DNA binding transcription factor activity
hydrolase activity, hydrolyzing O−glycosyl compounds
UDP−N−acetylmuramate dehydrogenase activity
oxidoreductase activity, acting on paired donors, with incorporation or reduction of molecular...
catalytic activity
heme binding
peroxidase activity
chromatin binding
iron ion binding
sequence−specific DNA binding
cellulose synthase (UDP−forming) activity
transporter activity
protein serine/threonine kinase activity
membrane
apoplast
extracellular region
transmembrane transport
protein phosphorylation
cellulose biosynthetic process
regulation of transcription, DNA−templated
metabolic process
carbohydrate metabolic process
response to oxidative stress
oxidation−reduction process
GO Terms
0
300
600
900
1200
DEGs Number in each GoTerm
Type
BP
CC
MF

G

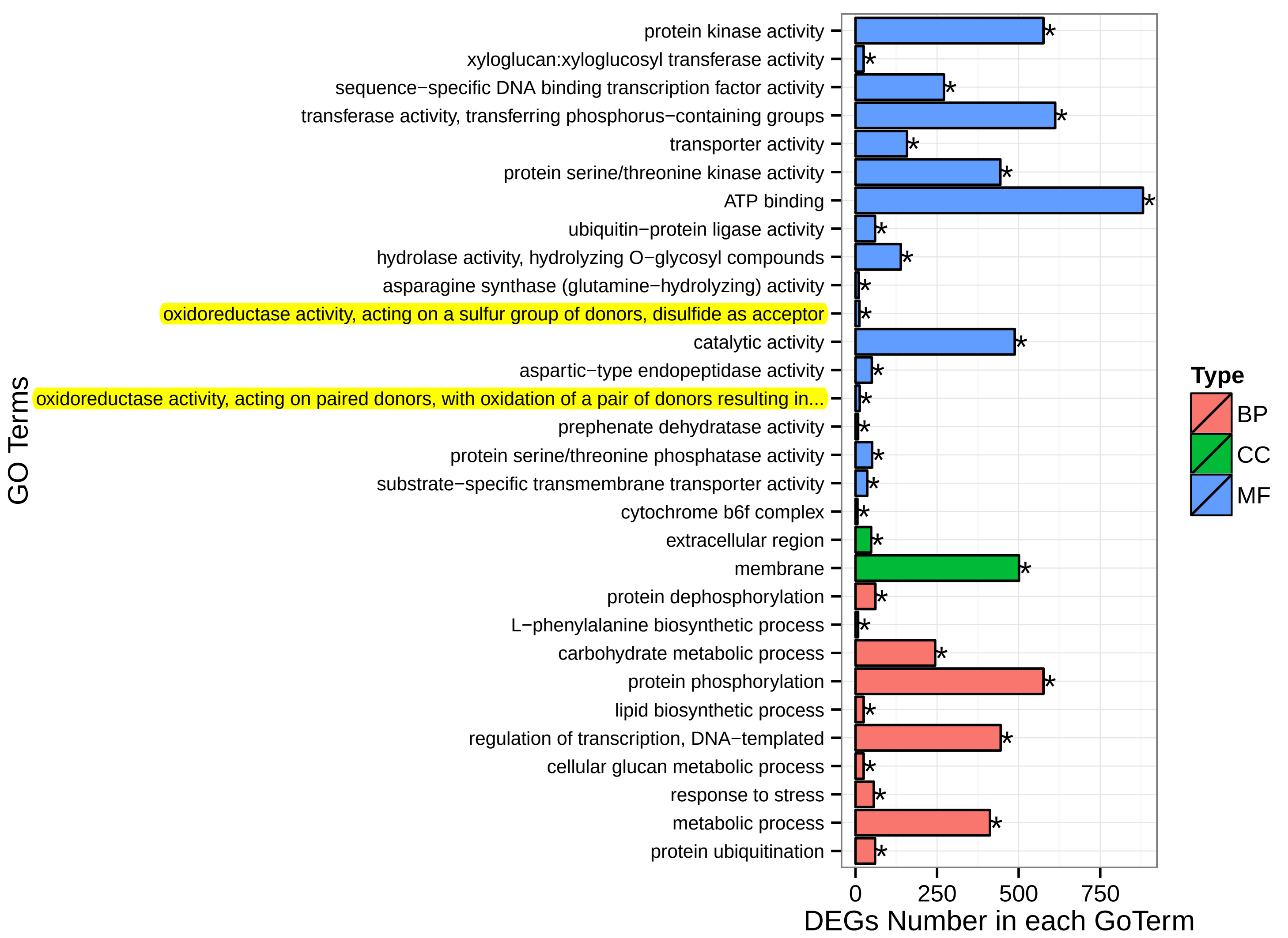
protein kinase activity
xyloglucan:xyloglucosyl transferase activity
sequence−specific DNA binding transcription factor activity
transferase activity, transferring phosphorus−containing groups
transporter activity
protein serine/threonine kinase activity
ATP binding
ubiquitin−protein ligase activity
hydrolase activity, hydrolyzing O−glycosyl compounds
asparagine synthase (glutamine−hydrolyzing) activity
oxidoreductase activity, acting on a sulfur group of donors, disulfide as acceptor
catalytic activity
aspartic−type endopeptidase activity
oxidoreductase activity, acting on paired donors, with oxidation of a pair of donors resulting in...
prephenate dehydratase activity
protein serine/threonine phosphatase activity
substrate−specific transmembrane transporter activity
cytochrome b6f complex
extracellular region
membrane
protein dephosphorylation
L−phenylalanine biosynthetic process
carbohydrate metabolic process
protein phosphorylation
lipid biosynthetic process
regulation of transcription, DNA−templated
cellular glucan metabolic process
response to stress
metabolic process
protein ubiquitination
GO Terms
0
250
500
750
DEGs Number in each GoTerm
Type
BP
CC
MF

H

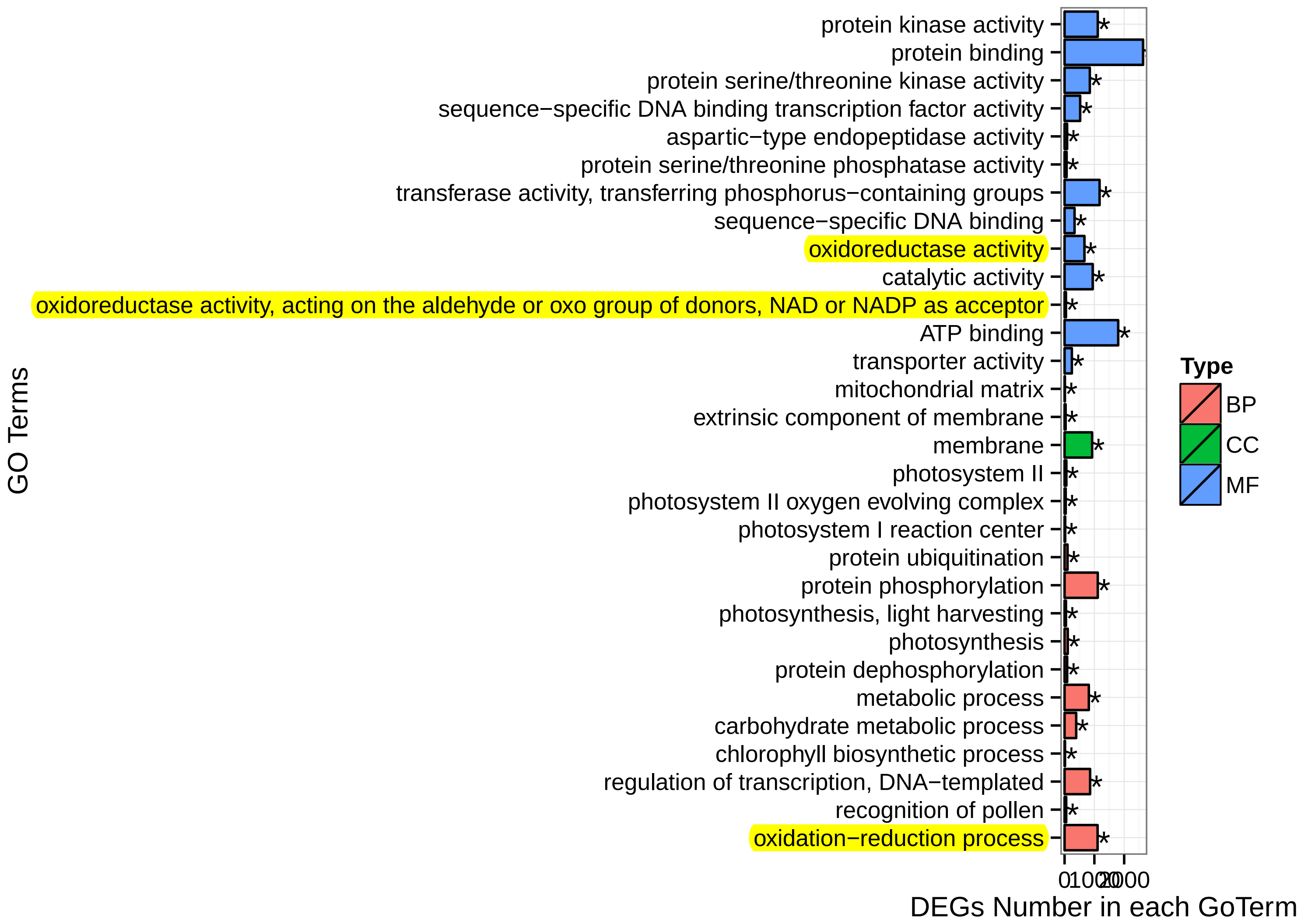

protein kinase activity
protein binding
protein serine/threonine kinase activity
sequence−specific DNA binding transcription factor activity
aspartic−type endopeptidase activity
protein serine/threonine phosphatase activity
transferase activity, transferring phosphorus−containing groups
sequence−specific DNA binding
oxidoreductase activity
catalytic activity
oxidoreductase activity, acting on the aldehyde or oxo group of donors, NAD or NADP as acceptor
ATP binding
transporter activity
mitochondrial matrix
extrinsic component of membrane
membrane
photosystem II
photosystem II oxygen evolving complex
photosystem I reaction center
protein ubiquitination
protein phosphorylation
photosynthesis, light harvesting
photosynthesis
protein dephosphorylation
metabolic process
carbohydrate metabolic process
chlorophyll biosynthetic process
regulation of transcription, DNA−templated
recognition of pollen
oxidation−reduction process
GO Terms
0
1000
2000
DEGs Number in each GoTerm
Type
BP
CC
MF

I

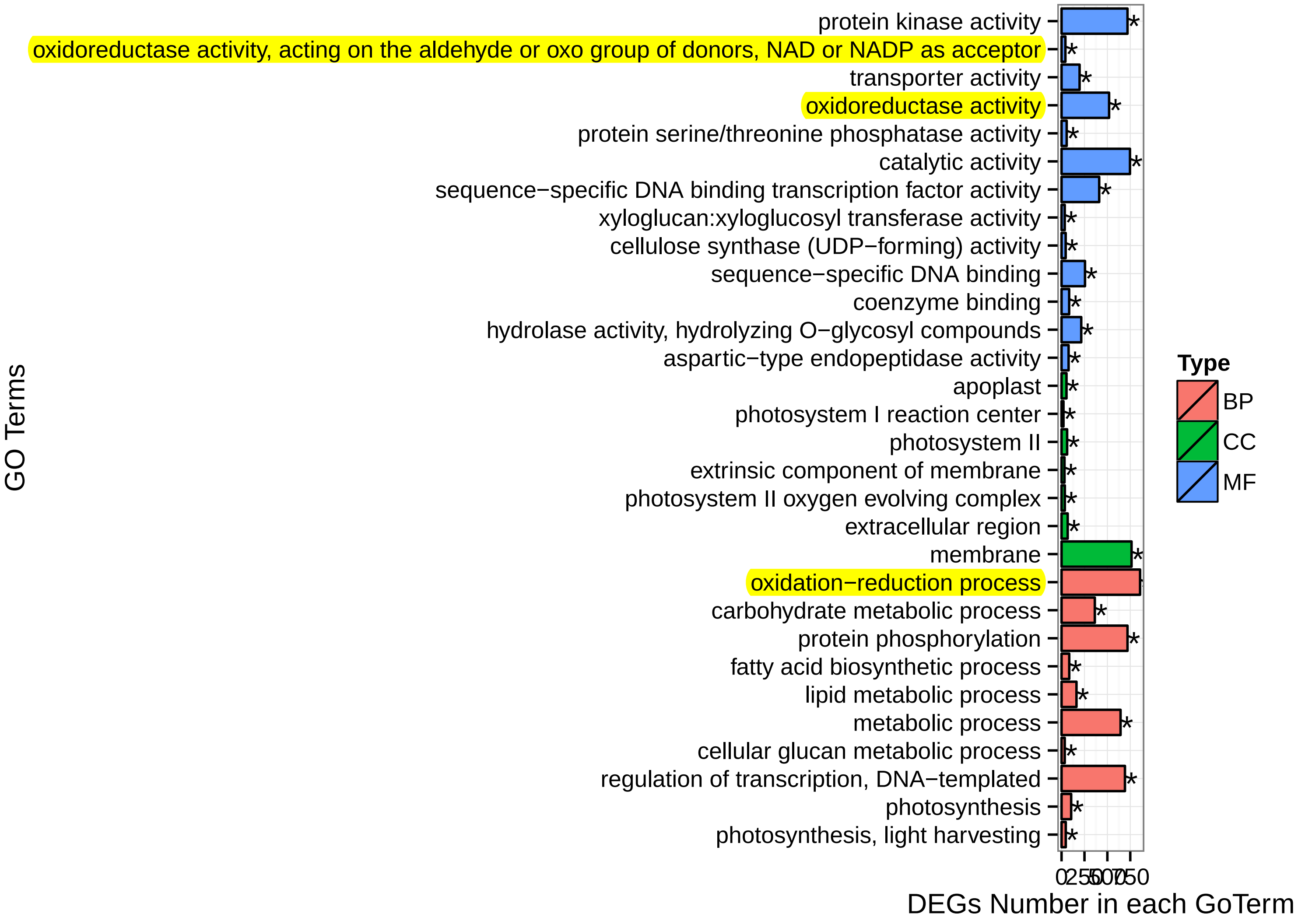

protein kinase activity
oxidoreductase activity, acting on the aldehyde or oxo group of donors, NAD or NADP as acceptor
transporter activity
oxidoreductase activity
protein serine/threonine phosphatase activity
catalytic activity
sequence−specific DNA binding transcription factor activity
xyloglucan:xyloglucosyl transferase activity
cellulose synthase (UDP−forming) activity
sequence−specific DNA binding
coenzyme binding
hydrolase activity, hydrolyzing O−glycosyl compounds
aspartic−type endopeptidase activity
apoplast
photosystem I reaction center
photosystem II
extrinsic component of membrane
photosystem II oxygen evolving complex
extracellular region
membrane
oxidation−reduction process
carbohydrate metabolic process
protein phosphorylation
fatty acid biosynthetic process
lipid metabolic process
metabolic process
cellular glucan metabolic process
regulation of transcription, DNA−templated
photosynthesis
photosynthesis, light harvesting
GO Terms
DEGs Number in each GoTerm
Type
BP
CC
MF

J

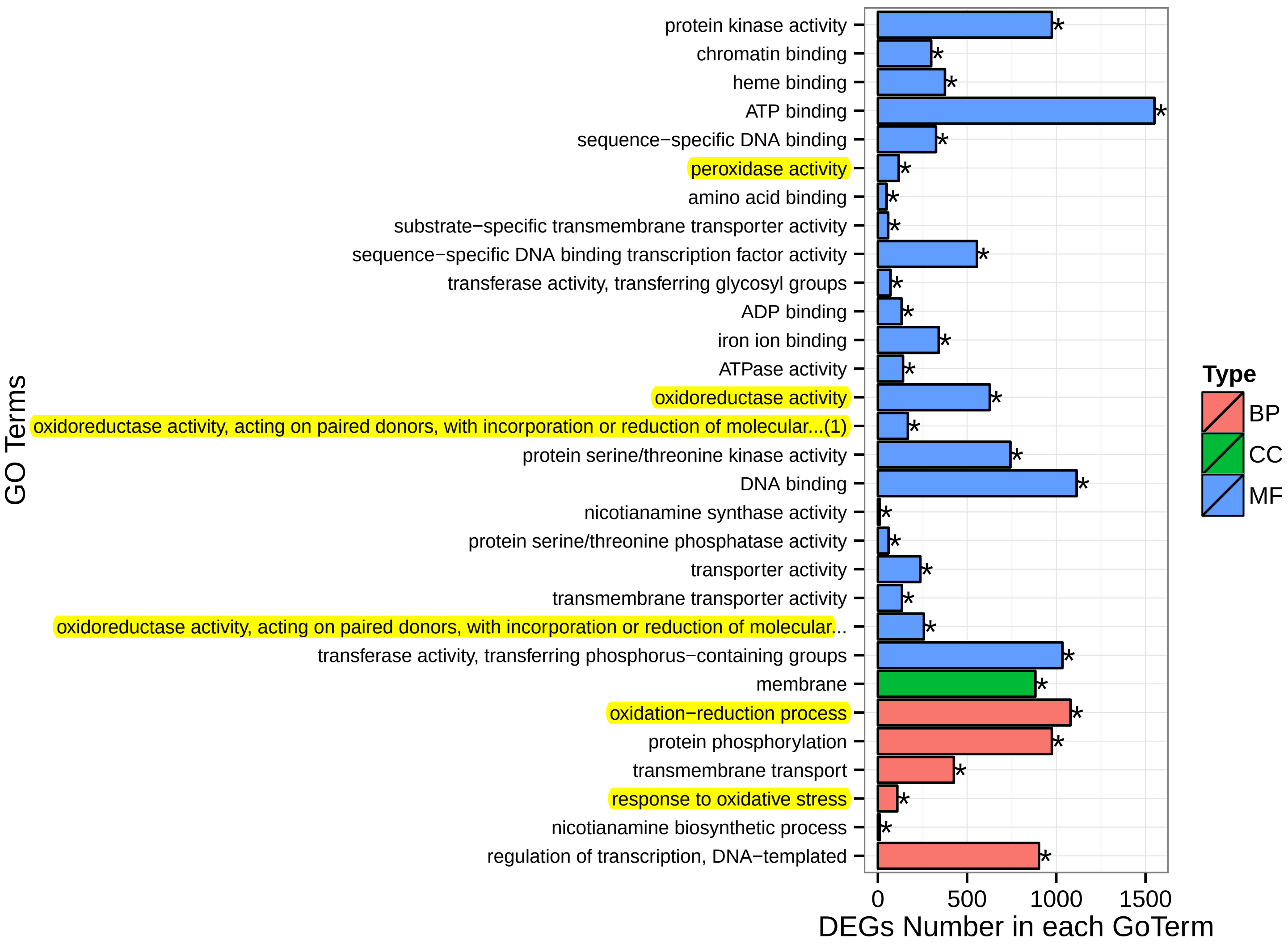
protein kinase activity
chromatin binding
heme binding
ATP binding
sequence−specific DNA binding
peroxidase activity
amino acid binding
substrate−specific transmembrane transporter activity
sequence−specific DNA binding transcription factor activity
transferase activity, transferring glycosyl groups
ADP binding
iron ion binding
ATPase activity
oxidoreductase activity
oxidoreductase activity, acting on paired donors, with incorporation or reduction of molecular...(1)
protein serine/threonine kinase activity
DNA binding
nicotianamine synthase activity
protein serine/threonine phosphatase activity
transporter activity
transmembrane transporter activity
oxidoreductase activity, acting on paired donors, with incorporation or reduction of molecular...
transferase activity, transferring phosphorus−containing groups
membrane
oxidation−reduction process
protein phosphorylation
transmembrane transport
response to oxidative stress
nicotianamine biosynthetic process
regulation of transcription, DNA−templated
GO Terms
0
500
1000
1500
DEGs Number in each GoTerm
Type
BP
CC
MF

K

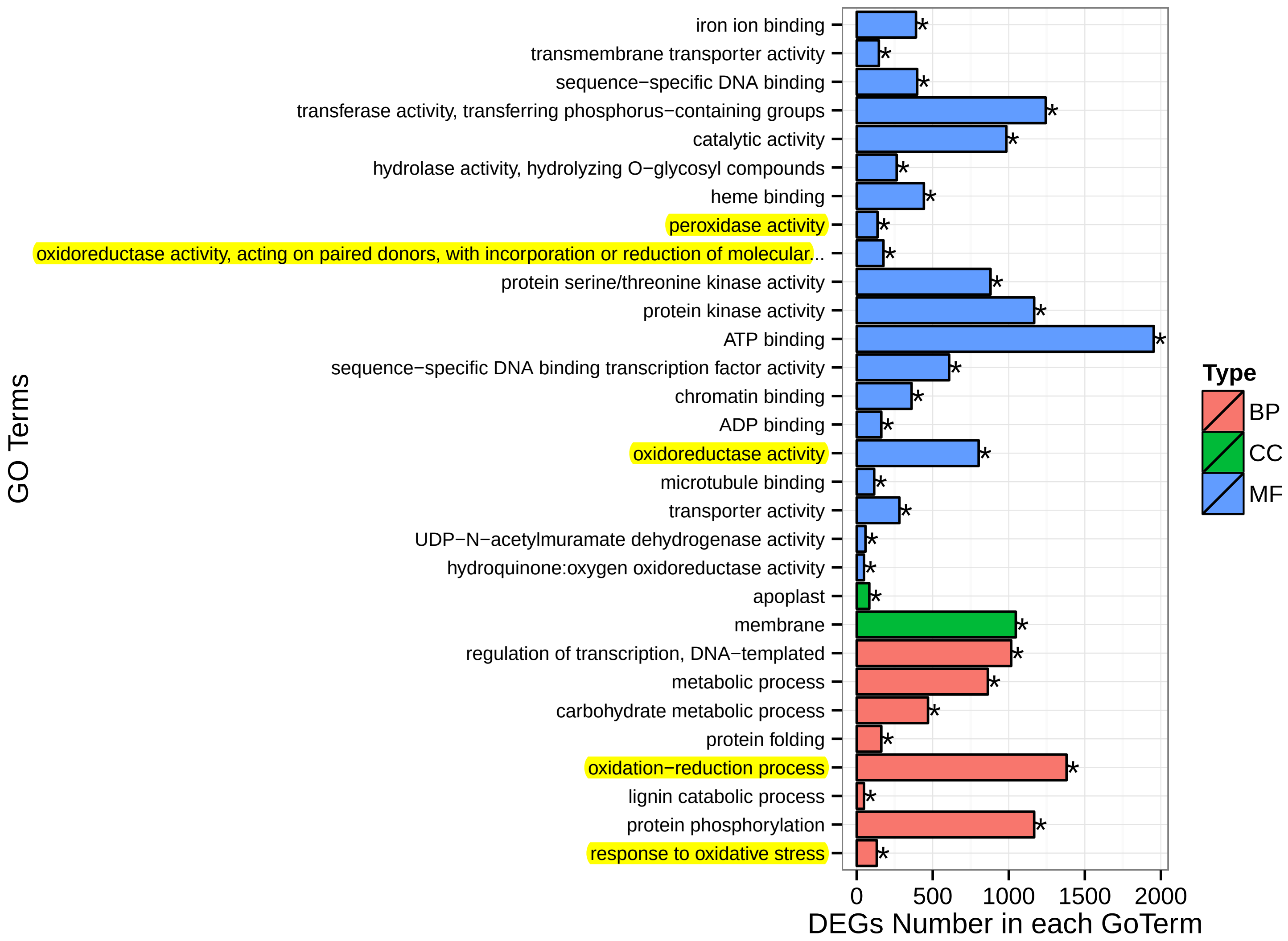

iron ion binding
transmembrane transporter activity
sequence−specific DNA binding
transferase activity, transferring phosphorus−containing groups
catalytic activity
hydrolase activity, hydrolyzing O−glycosyl compounds
heme binding
peroxidase activity
oxidoreductase activity, acting on paired donors, with incorporation or reduction of molecular...
protein serine/threonine kinase activity
protein kinase activity
ATP binding
sequence−specific DNA binding transcription factor activity
chromatin binding
ADP binding
oxidoreductase activity
microtubule binding
transporter activity
UDP−N−acetylmuramate dehydrogenase activity
hydroquinone:oxygen oxidoreductase activity
apoplast
membrane
regulation of transcription, DNA−templated
metabolic process
carbohydrate metabolic process
protein folding
oxidation−reduction process
lignin catabolic process
protein phosphorylation
response to oxidative stress
GO Terms
0
500
1000
1500
2000
DEGs Number in each GoTerm
Type
BP
CC
MF

L

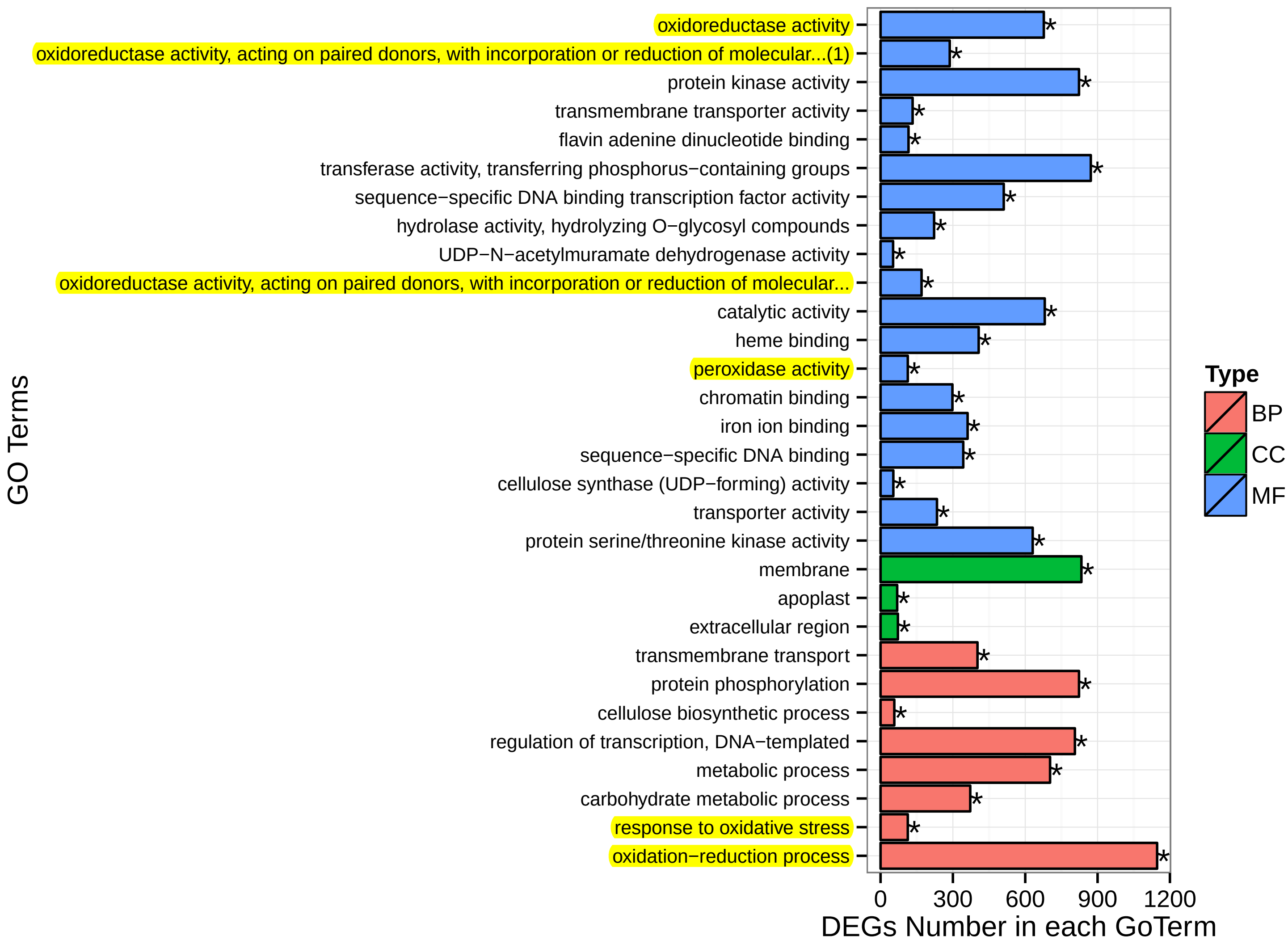
oxidoreductase activity
oxidoreductase activity, acting on paired donors, with incorporation or reduction of molecular...(1)
protein kinase activity
transmembrane transporter activity
flavin adenine dinucleotide binding
transferase activity, transferring phosphorus−containing groups
sequence−specific DNA binding transcription factor activity
hydrolase activity, hydrolyzing O−glycosyl compounds
UDP−N−acetylmuramate dehydrogenase activity
oxidoreductase activity, acting on paired donors, with incorporation or reduction of molecular...
catalytic activity
heme binding
peroxidase activity
chromatin binding
iron ion binding
sequence−specific DNA binding
cellulose synthase (UDP−forming) activity
transporter activity
protein serine/threonine kinase activity
membrane
apoplast
extracellular region
transmembrane transport
protein phosphorylation
cellulose biosynthetic process
regulation of transcription, DNA−templated
metabolic process
carbohydrate metabolic process
response to oxidative stress
oxidation−reduction process
GO Terms
0
300
600
900
1200
DEGs Number in each GoTerm
Type
BP
CC
MF
