# Supplementary material for: Deep Transcriptome Analysis Reveals Reactive Oxygen Species (ROS) Network Evolution, Response to Abiotic Stress, and Regulation of Fiber Development in Cotton
Source: Int J Mol Sci. 2019 Apr 15;20(8):1863. doi: 10.3390/ijms20081863 (PMC6514600; doi:10.3390/ijms20081863)
Supplement: Supplementary file 1 [file ijms-20-01863-s001.zip › Supplementary files/Table S9.docx]

Table S9: List of primers used for qRT-PCR.

| Gene ID | **Gene Name** | **Forward Primer(5'-3')** | **Reverse Primer(5'-3')** |
| --- | --- | --- | --- |
| Gh_A03G1663 | *GhTrx15_At* | ATGCCCCTGTTCTTGTCGAG | CACCGGTACGGCCATAATGT |
| Gh_A05G2211 | *GhRBOH11_At* | ACCATTGGTCTTAGGTGGCG | CCATGGTGATTTCCGGTGGA |
| Gh_A09G2473 | *GhCSD3_At* | TACGAGAGCACCGAGGAAGA | TGGAACGAAGGGAAAGACCG |
| Gh_A02G0651 | *GhPER61_At* | GATCTGTGTTGCTGGACGGA | TAGGTTCTGCAATGTGGCGT |
| Gh_A09G1501 | *GhDHAR4_At* | GGTGCCGTCCTTGGAACATA | TAGGCATCGATCTTGTCCGC |
| Gh_D05G2811 | *GhTrx25_Dt* | GGATGATGCGCCATTGACAG | ACAAGGTTGTGCGGAACAGT |
| Gh_D05G0244 | *GhPrxR6_Dt* | GGCCAAAACTTACCCGGAGA | GAGCGAACCTCTTGGAACGA |
| Gh_D12G2750 | *GhRBOH3_Dt* | TATCCGCTTCACGATCACCG | GAAGTGCTTTATGAGCGCCG |
| Gh_D05G1507 | *GhGLR43_Dt* | TTGGTTGTGGAGAATGCGGT | ACTCCACCCCCGTTTGTTAC |
| Gh_D03G0246 | *GhPER20_Dt* | CGCAACTATCCGCCTCTTCT | GCAATGACATCACGAGTCGC |
